# Injection lipolysis in aesthetic medicine: mechanisms, clinical safety, and clinical applications

**DOI:** 10.3389/fmed.2026.1865071

**Published:** 2026-07-21

**Authors:** Yinjie Ao, Yingjin Zhou

**Affiliations:** Department of Plastic Surgery and Medical Cosmetic Center, The Affiliated Eye Hospital, Jiangxi Medical College, Nanchang University, Nanchang, China

**Keywords:** complication management, deoxycholic acid, injection lipolysis, mechanisms, precision fat reduction

## Abstract

**Background:**

Injection lipolysis is increasingly used as a minimally invasive option for focal fat reduction. However, systematic data on its safety and efficacy remain limited.

**Objective:**

This review summarizes the development, mechanism, clinical application, complication management, and future directions of injection lipolysis, grounded in the anatomy of facial and cervical fat.

**Methods:**

We searched PubMed for literature published between 1943 and 2026 to trace the research history of lipolytic agents. *In vitro* data were used to compare the differential effects of deoxycholic acid (DCA) on adipocytes versus neural tissue. Clinical indications, injection techniques, and complication strategies were also reviewed.

**Results:**

Deoxycholic acid preferentially disrupts adipocytes and exerts cytolytic effects with relative tissue selectivity. The submental fat pad is the best-documented indication. Poor injection technique can lead to skin necrosis, marginal mandibular nerve palsy, and other complications.

**Conclusion:**

Injection lipolysis is an effective tool for spot fat reduction, but its safety depends heavily on anatomical knowledge, patient selection, and strict procedural discipline. Future work should focus on precision fat reduction and further clinical validation.

## 1 Introduction

Localized fat accumulation is a common complaint in outpatient clinics. These patients typically have a normal body mass index but present with discrete fat bulges in areas such as the submental region, cheeks, or abdomen that do not respond well to diet or exercise (1). Surgical liposuction is effective but carries risks of trauma, prolonged recovery, and possible skin laxity or contour irregularities. This has driven clinical interest in minimally invasive fat reduction techniques (2). Injection lipolysis emerged in this context. Three compliant deoxycholic acid (DCA)-based products are currently available worldwide: Kybella (Allergan, FDA-approved in 2015), V-OLET (Daewoong Pharmaceutical, approved in South Korea in 2021), and Rongzhi^®^ (Minovapharma, approved by NMPA in June 2025). All three are indicated for moderate to severe submental fat convexity in adults. Before these approvals, most so-called “lipolysis injections” on the Chinese market came from unofficial channels or were misused cosmetic- or device-grade products (3). Safety risks were high, and adverse events such as skin necrosis, infection, and contour deformities were common. The availability of regulatory-approved products now provides a foundation for standardization in non-surgical fat reduction (4).

This review, based on our clinical experience and the literature, examines the mechanism, indications, injection techniques, and complication management of injection lipolysis from an anatomical perspective, and discusses its future directions and limitations with reference to currently approved products.

## 2 Methods

We searched PubMed for literature published between 1943 and 2026 using combinations of the following keywords: “deoxycholic acid,” “injection lipolysis,” “submental fat,” “phosphatidylcholine,” “adipocyte,” Cytolytic cell death and “complication.” We included clinical studies, mechanistic studies, case reports, and systematic reviews related to injection lipolysis. Non-English articles, conference abstracts, and studies not involving deoxycholic acid or human/*in vitro* fat models were excluded. The initial search yielded 342 articles. After screening titles and abstracts, 89 full-text articles were assessed for eligibility, and 57 were finally included in this review. Data synthesis was performed narratively, organized by themes: mechanism, clinical application, complications.

## 3 Anatomy of facial and cervical fat: clinical relevance

Facial and cervical fat is divided by the SMAS fascia. Deep compartments and superficial fat pads. Injection lipolysis targets the superficial fat pads (subcutaneous fat). These pads are not completely separated by fibrous septa, allowing some drug diffusion to adjacent areas (5). Subcutaneous fat can be further divided into superficial and deep layers. The superficial layer lies just beneath the dermis, contains rich capillary networks, and provides nutritional support to hair follicles (6). The deep layer lies between the superficial fat and SMAS, is structurally looser, and is the primary site of fat accumulation (7). From an applied anatomical perspective, the target for injection lipolysis is the deep layer of the superficial fat pad. Injecting too superficially risks damaging blood vessels, increasing bruising and skin necrosis, and potentially compromising hair follicle blood supply (8). The deep layer has more pronounced fat accumulation, allows more homogenous drug distribution, offers better lipolytic efficiency, and is farther from the skin surface, improving safety. Cervical fat requires further distinction. The target is subcutaneous fat superficial to the platysma (the main body of the submental fat pad), where fat accumulation is most evident and is the primary target for contouring (9). Injection into the deep platysma layer must be strictly avoided to protect important structures such as the marginal mandibular branch of the facial nerve (10). Understanding these anatomical layers is essential for planning injection depth and preventing skin necrosis and nerve injury.

## 4 History and mechanism of injection lipolysis

The concept of injection lipolysis dates back to the mid-20th century (11). Phosphatidylcholine (PC), used as an intravenous fat emulsifier for hyperlipidemia, was observed to cause fat atrophy at injection sites, sparking interest in its use for local fat reduction (12). Since then, PC-based “lipolysis injections,” often combined with DCA or other excipients, have appeared on the aesthetic market (13). Most were office-formulated or small-batch products lacking standardized quality control and prospective clinical trials, with insufficient safety data (13). The real turning point came with the introduction of deoxycholic acid (DCA), an endogenous bile acid that enhances adipocyte membrane disruption when combined with PC (14). The move from PC-DCA combinations to DCA monotherapy was largely motivated by concerns over safety and standardization. Compounded mixtures often lacked consistent composition and quality control, whereas DCA monotherapy offers a purified, single-component formulation. In 2015, Allergan’s DCA monotherapy (Kybella) received FDA approval for moderate to severe submental fat convexity, becoming the first officially approved injection lipolysis product worldwide (15). A PubMed-based bibliometric analysis shows that research on lipolytic agents in medicine began in 1943 (83 years of cumulative work by 2026), fat-related research began in 1967 (59 years), but dedicated research on “injection lipolysis” began only in 2014 (12 years by 2026) (16–18). This indicates that while the biological effects of lipolytic agents have long been recognized, their translation into standardized injectable products with systematic clinical research has accelerated only in the past decade. Subsequently, Daewoong Pharmaceutical’s V-OLET (DCA monotherapy) was approved in South Korea in 2021 and later entered China via the Hainan Boao (19). In June 2025, Minovapharma Rongzhi^®^ (DCA monotherapy) received NMPA approval. Thus, three compliant DCA-based injection lipolysis products are now approved in the United States, South Korea, and China, marking a fundamental shift from empirical use to standardized treatment (Table 1).

**TABLE 1:** Timeline of injection lipolysis development.

Year	Event	Significance
1943	First use of phosphatidylcholine (PC) for hyperlipidemia research	Discovery of PC’s lipid-emulsifying properties
1967	Deoxycholic acid (DCA) introduced for localized fat-related research	Established foundation for lipolytic agents
2000s	PC/DCA combination products used off-label in European and US clinics	Clinical demand emerged, but evidence was lacking
2010	REFINE phase III RCT program for ATX-101 initiated	High-level evidence began to accumulate
April 2015	FDA approval of ATX-101 (Kybella^®^)	First approved injection lipolysis product worldwide
2021	Approval of V-OLET^®^ (Daewoong Pharmaceutical) in South Korea	First approved product in Asia
June 2025	NMPA approval of Rongzhi^®^ (Minovapharma) in China	First approved injection lipolysis product in China

### 4.1 Core component and mechanism of action

**FIGURE 1:**
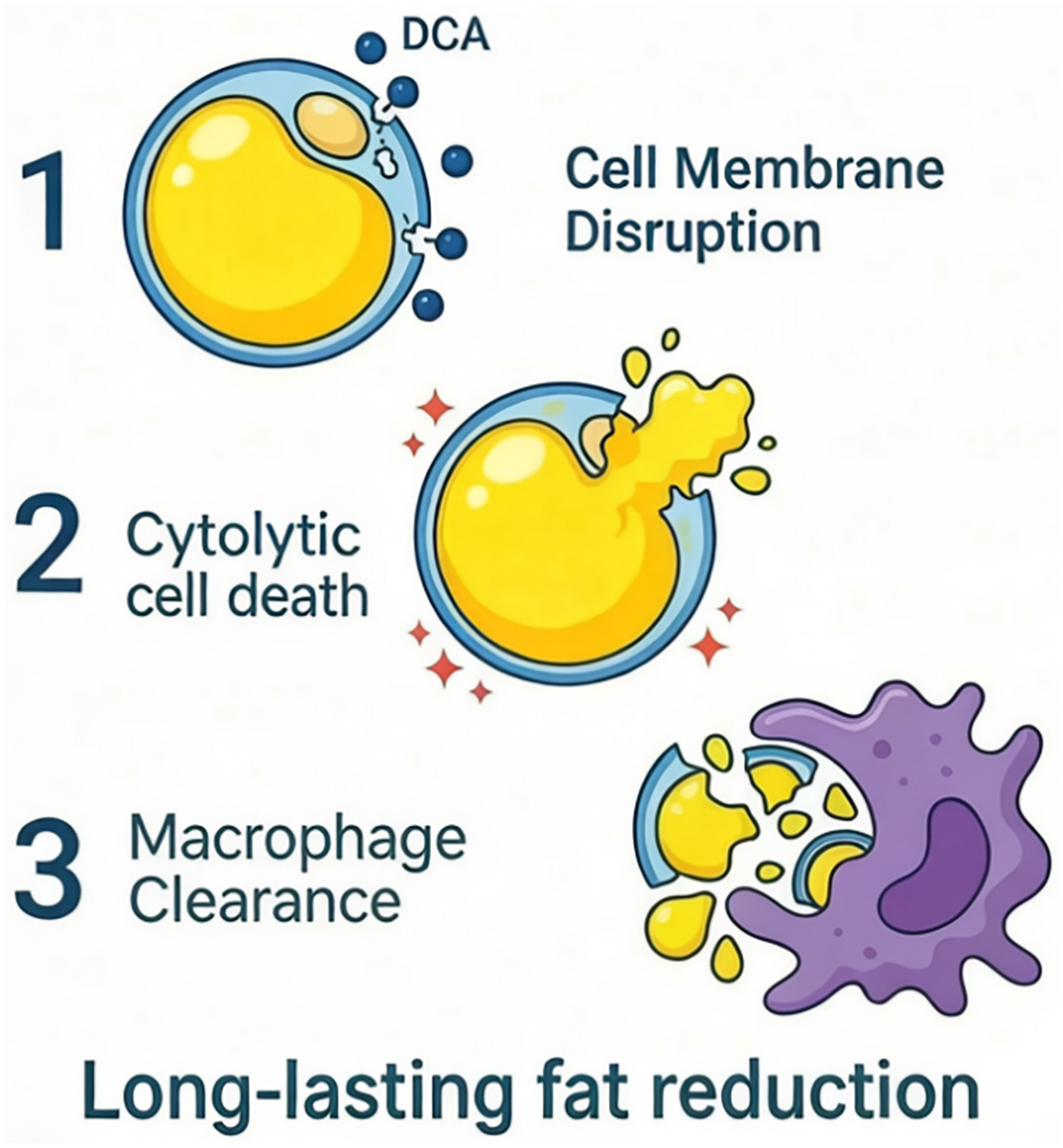
Long-lasting fat reduction.

Deoxycholic acid (DCA) is an endogenous secondary bile acid. Its lipolytic mechanism works as follows: acting as a non-ionic surfactant, DCA inserts into the phospholipid bilayer of the adipocyte membrane, alters membrane permeability, and causes cell membrane rupture and lysis (20) (Figure 1). Importantly, DCA induces Cytolytic cell death, not apoptosis. Cytolytic cell death is an inflammatory form of programmed cell death characterized by pore formation, cell swelling and rupture, and release of intracellular contents, which triggers a strong inflammatory response (21). Unlike apoptosis, Cytolytic cell death causes irreversible cell destruction. The released free fatty acids and triglycerides are then cleared by macrophages, resulting in an irreversible reduction in adipocyte number (22). DCA has relative tissue selectivity. *In vitro* data show that within 20 min of exposure, DCA causes visible membrane damage to adipocytes, with significant inflammation by 24 h (23). It also affects myelin and neural tissue, but the damage is milder and reversible. This provides a theoretical basis for why nerve complications such as marginal mandibular nerve palsy typically resolve spontaneously within 4–8 weeks (24). In saturation attack experiments under extreme dose conditions, DCA-induced damage remains largely confined to adipocytes, with limited injury to surrounding non-target tissues. Rongzhi^®^ is a deoxycholic acid-based product recently approved in China. Its formulation contains no benzyl alcohol. Benzyl alcohol is a common preservative and local anesthetic in injectable preparations, but studies suggest it may worsen local tissue damage, prolong inflammation, and contribute to adverse reactions such as induration and granuloma (25). The absence of benzyl alcohol in Rongzhi theoretically reduces non-target tissue irritation and postoperative local reactions, representing a formulation-level improvement.

## 5 Current clinical use of injection lipolysis

### 5.1 Established indication: submental fat

Submental fat (double chin) is the only indication approved by major regulatory bodies such as the FDA and NMPA (26, 27). Fat accumulation in this area is common even in individuals with normal BMI and responds poorly to diet or exercise. Multicenter, randomized, double-blind, placebo-controlled phase III trials confirmed that DCA monotherapy meets both efficacy and safety endpoints, with significant fat reduction and high patient satisfaction. All three products carry this as their core approved label (28).

### 5.2 Off-label uses

Although approved indications are limited to submental fat, injection lipolysis is sometimes used off-label for other areas of localized fat. It is important to note that the FDA label for Kybella states that safe and effective use outside the submental region has not been established. Therefore, clinicians considering off-label applications should exercise particular caution, obtain thorough informed consent, and strictly adhere to safety protocols.

Face. The buccal fat pad (“baby fat”), oral commissure pouches, and mandibular border fat are common concerns (29). Injection around the buccal fat pad should avoid the parotid duct and buccal branches of the facial nerve. For the oral commissure pouches, dose control is critical to prevent overcorrection leading to depressions or skin laxity (30). The mandibular border requires particular caution due to its proximity to the marginal mandibular nerve. Injection depth should be strictly limited to the deep layer of the superficial fat pad (31). Neck. Platysmal bands and cervical fat pads (superficial to the platysma) are common targets. The skin here is thin, and improper depth increases the risk of necrosis or depressions (32). McKay et al. recommended that pinch the skin with the non-dominant hand and confirm needle position in the subcutaneous fat layer before injecting, avoid penetrating the platysma (33). Other body areas. The abdomen, flanks, arms, and back are occasionally treated (34, 35). Fat layers are thicker here, offering more room to work, but large treatment areas and higher total doses lead to more pronounced postoperative swelling and induration. High-quality clinical trials supporting body contouring use are lacking, efficacy and safety data come mainly from case reports and small case series (Table 2).

**TABLE 2:** Key anatomical structures and safety considerations for facial and cervical injection lipolysis.

Anatomical region	Key structures	Injection safety
Submental region	Submental fat pad, platysma	Target subcutaneous fat superficial to platysma; avoid deep injection into submental vessels
Mandibular border	Marginal mandibular nerve	Strictly inject in subcutaneous fat layer; avoid high-dose injection along nerve course
Jowl	Mandibular ligament, depressor anguli oris	Inject in deep subcutaneous fat layer; avoid overcorrection
Buccal area	Buccal fat pad, parotid duct	Avoid parotid duct area (approximately 1.5–2.0 cm below zygomatic arch); avoid deep injection into buccal fat pad
Parotid duct region	Parotid duct	Identify duct trajectory before injection; avoid high-dose injection in this area
Facial nerve branches	Temporal, zygomatic, buccal, marginal mandibular, cervical branches	Keep injection in subcutaneous fat layer; blunt cannula reduces nerve injury risk
Platysma	Platysma muscle, retroplatysmal fat	Target subcutaneous fat superficial to the platysma; penetrating platysma risks deep neurovascular injury
Vascular danger zones	Facial artery, submental artery, superficial temporal artery, supratrochlear artery	Aspirate before injection; avoid high-pressure or high-volume injection; be familiar with vessel course

### 5.3 Clinical evidence of DCA injection

Clinical data on DCA injection for various anatomical sites remain fragmented. For the neck and submental area, a 12-months study of 165 patients receiving up to 6 sessions of ATX-101 (2 mg/cm^2^) reported ≥1-grade improvement in 86.8% (clinician-assessed) and 83.8% (patient-assessed) at 12 weeks, with 84.9% satisfied at 12 months (9). For jowls, a prospective study of 12 patients injected DCA subcutaneously within a circular area 1.0 cm above the mandibular border, with response assessed by GAIS (36). For the brassiere line, five patients received three sessions of Kybella (2 mg/cm^2^, 0.2 mL per point at 1-cm intervals) at 4-weeks intervals; two additional cases using 0.15 mL per point (0.5–1.0 cm apart) reported gradual fat reduction from 1 month to 9 months (37). For the lower abdomen, a study of 11 patients treated with 8.88 mL per side over 2–4 sessions at 8-weeks intervals reported significant reductions in anterior (32.8 ± 4.0 to 28.7 ± 3.4 cm, *p* = 0.04) and lateral (24.0 ± 4.1 to 21.7 ± 4.0 cm, *p* < 0.01) subcutaneous fat thickness (38, 39). Collectively, these findings demonstrate that DCA injection produces noticeable fat reduction across all treated areas. However, considerable heterogeneity in dosing, treatment intervals, and assessment methods across these studies precludes the establishment of a unified protocol at present (Table 3).

**TABLE 3:** Summary of clinical data on DCA injection for anatomical sites.

Anatomical site	Study design	Patient population	Treatment regimen	Key outcomes
Neck/submental	12-months open-label study	165 patients with moderate-to-severe SMF	Up to 6 sessions of ATX-101 (2 mg/cm^2^)	≥1-grade improvement: 86.8% (clinician)/83.8% (patient) at 12 weeks; 84.9% satisfied at 12 months (9)
Jowls	Prospective study	12 patients with mild-to-moderate jowl fat	DCA injected subcutaneously within a circular area 1.0 cm above the mandibular border	Response assessed by physician- and subject-rated GAIS (36)
Brassiere line	Case series	5 patients (posterior bra-bulge)	3 sessions of Kybella (2 mg/cm^2^, 0.2 mL per point, 1-cm intervals) at 4-weeks intervals	Gradual fat reduction observed (37)
Brassiere line	Case report	2 patients	0.15 mL per point (0.5–1.0 cm apart)	Gradual fat reduction from 1 month to 9 months (37)
Lower abdomen	Clinical study	11 patients	8.88 mL per side, 2–4 sessions at 8-weeks intervals	Significant reduction in anterior (*p* = 0.04) and lateral (p < 0.01) subcutaneous fat thickness (38, 39)

## 6 Complications and risk management

### 6.1 Common adverse reactions

Most adverse reactions are transient and resolve without specific intervention.

Immediate post-injection. Pain, burning sensation, edema, and bruising are the most common. Pain and burning usually subside within minutes to hours (9). Edema peaks at 24–48 h and lasts 3–7 days. Bruising typically resolves within 1–2 weeks (40). Immediate post-procedure ice application helps reduce these reactions. Subacute phase. Induration and nodules are relatively common later reactions, with incidence varying by dose and individual factors. They result from fibrous tissue proliferation after local fat necrosis and usually soften and resolve within 4–8 weeks (22). Local depressions are often caused by overly superficial injection or uneven dosing; some improve over time, but severe cases may persist (41).

### 6.2 Serious complications

Serious complications are rare but can cause significant functional or cosmetic damage when they occur. Skin necrosis and ulceration. Main causes include overly superficial injection (drug entering the dermis), excessive dose per point causing high local pressure, and accidental intravascular injection leading to embolic necrosis (19). Clinical signs include local pallor and cyanosis within days, followed by blistering, crusting, and ulceration. Commonly occurs in areas with relatively poor blood supply, such as the mandibular border and submental region (42). Nerve injury. Marginal mandibular nerve palsy is the most concerning nerve complication, presenting as ipsilateral mouth droop and depressor anguli oris dysfunction (43). Prevention focuses on strict control of injection area and depth, avoiding high-dose injection along the nerve’s course. Infection and abscess. Usually related to poor aseptic technique or improper post-procedure care. Presents with worsening redness, pain, and warmth at the injection site, abscess may form in severe cases. Requires prompt antibiotics, drainage if abscess develops (44). Scar hypertrophy. Relatively rare, typically seen in patients who develop skin necrosis after injection or those with a history of keloids (45). Presents as raised, firm, erythematous scar tissue beyond the original injection area. Prevention focuses on avoiding skin necrosis.

### 6.3 Prevention and management of complications

Pre-procedural assessment and contraindications. Take a detailed medical history. Exclude contraindications such as autoimmune disease, coagulation disorders, active local infection, pregnancy, and breastfeeding (46). Patients with a history of keloids or hypertrophic scarring should be fully informed of the risks. Take pre-procedure photographs for later comparison. Intra-procedural technique. Control the dose per injection point and total dose per session within safe limits for the area. Aspirate before each injection to rule out intravascular placement (47). The target depth is the deep layer of the superficial fat pad. A needle of 4–6 mm is generally adequate for most facial areas; a practical guide is to insert the full needle tip subcutaneously then withdraw slightly before injecting. Avoid concentrated injection along the course of the marginal mandibular nerve. Post-procedure care and follow-up. Apply ice packs immediately after injection for about 15–20 min. Reassure patients that post-procedure edema and induration are typically transient and self-limiting (23). Gentle massage within the first week to 10 days may help soften induration. Advise patients to return for evaluation if they experience unusual pain, skin color changes, or fever (48).

## 7 Discussion

A few points from this review are worth highlighting. Efficacy is not the same as safety. DCA has demonstrated clear effectiveness for submental fat in clinical trials, but off-label use on the face, neck, or body rests on much thinner evidence (49). These areas have more complex anatomy and higher risks. Clinicians who choose off-label applications should proceed with caution, obtain thorough informed consent, and adhere to rigorous injection technique. Over-treatment with any non-surgical fat reduction modality–whether DCA, radiofrequency, or cryolipolysis–can lead to excessive subcutaneous fibrosis (50). Fibrosis is not merely a cosmetic issue causing induration or contour irregularities, it also disrupts normal tissue planes and complicates future surgical procedures such as neck lifts (51). Respecting tissue healing time and avoiding overly aggressive protocols are therefore essential. The availability of compliant DCA products in China, the United States, and South Korea marks an important step toward standardization. However, non-regulatory-approved products remain a persistent safety threat. Regulatory efforts should extend beyond product approval to include operator training, complication reporting systems, and clear practice guidelines (52). Finally, injection lipolysis should not be viewed as a standalone “inject and forget” procedure. Prospective studies are needed to validate these combination strategies.

## 8 Summary

It is important to distinguish between efficacy and safety when considering injection lipolysis. DCA injection lipolysis effectively reduces focal fat through adipocyte Cytolytic cell death, with phase III trials confirming its efficacy for submental fat. However, its safety depends heavily on anatomical knowledge, patient selection, and procedural discipline. Safety and outcomes, however, depend on anatomical knowledge, patient selection, and procedural discipline. Off-label use requires caution to avoid excessive fibrosis that may compromise future surgery. Regulatory-approved products enable standardization, but clinical value ultimately rests on operator skill. The principle of “first, do no harm” should always guide practice.
